# Efficacy and safety of Shen-Song-Yang-Xin capsule for treating arrhythmia in the elderly patients with coronary heart disease: Protocol for a systematic review and meta-analysis

**DOI:** 10.1097/MD.0000000000013599

**Published:** 2018-12-21

**Authors:** Zhicong Zeng, ZhenJie Zhuang, YingXian He, ZhaoJun Yang, Yinzhi Song

**Affiliations:** a Guangzhou University of Chinese Medicine, Guangzhou; b Baoan Hospital of Traditional Chinese Medicine in Shenzhen, Shenzhen, China.

**Keywords:** arrhythmia, coronary heart disease, protocol, Shen-Song-Yang-Xin capsule, systematic review

## Abstract

**Background:**

Coronary heart disease (CHD) is a major cause of mortality worldwide. Shen-Song-Yang-Xin capsule (SSYXC) has received extensive attention as an alternative therapy in improving myocardial ischemia and hypoxia effectively. In addition, there has been no systematic review or meta-analysis of SSYXC in the treatment of the elderly patients with cardiac arrhythmias in coronary heart disease (CHD). Therefore, we carry out a protocol of a proposed study based on the referred Reporting Items for Systematic Reviews and Meta-Analyses guidelines that aims to systematically evaluate the efficacy and safety of SSYXC in the elderly patients with cardiac arrhythmias in CHD.

**Methods:**

Two researchers will search 9 electronic databases (PubMed, Medline, Embase, Cochrane Library, Web of Science, China National Knowledge Infrastructure, Chinese VIP Information, Wanfang Database, and Chinese Biomedical Database) to identify all studies that meet the inclusion criteria and were published before October 2018. The literature selection process will be reported in accordance with the PRISMA guidelines. After information extraction and methodological quality evaluation, we will use Stata 12.0 software (STATA Corporation, College Station, TX) to synthesize the data. The primary outcomes will include effective rates of treatment and improvements of electrocardiogram or 24 hours dynamic electrocardiogram result, and secondary outcomes will include improvement of relevant serological indexes, heart function classification and adverse events.

**Results:**

The data synthesis results will objectively illustrate the efficacy and safety of SSYXC in the elderly patients with cardiac arrhythmias in CHD.

**Conculsion:**

The findings will provide a reference for the use of SSYXC in the treatment of the elderly patients with cardiac arrhythmias in CHD.

**Registration:**

PROS-PERO CRD42018112570.

## 1 Introduction

According to the World Health Organization (WHO) (2014), Coronary heart disease (CHD) is a major cause of mortality worldwide, causing more than 7 million people death every year.^[20]^

The end point of the development of CHD is sudden cardiac death (SCD) and heart failure, while one of the predisposing and progressive factors of SCD and heart failure is arrhythmia.^[16]^ Arrhythmia is commonly classified into several different clinical presentation including sinus tachycardia or bradycardia, atrial or ventricular premature complex, supraventricular or ventricular tachycardia, atrial flutter or fibrillation and ventricular fluter or fibrillation. According to the guidance of Scottish Intercollegiate Guidelines Network (SIGN) (2018), acute coronary syndrome (ACS), is closely related to the occurrence of atrial fibrillation (AF) and ventricular arrhythmia (VA).^[1]^ In addition, arrhythmias are much more frequent in patients with advanced age^[2]^ and, for example, AF occurs more commonly in those who are older.^[8]^ As for treating arrhythmia, Class I antiarrhythmic drugs (Na^+^ channel blockers), amiodarone, digoxin, beta blockade, calcium channel blockers (verapamil or diltiazem), and dronedarone are commonly used. However, those antiarrhythmic drugs (AAD) could reduce arrhythmic death but they has fewer overall benefit to total mortality of patient, which is important for the elderly patients. Moreover, patients with structural heart disease have higher risks of ventricular arrhythmia as well as higher risks of proarrhythmia after utilization of antiarrhythmic medications.^[7]^ For example, dronedarone should not be given to patients with left ventricular systolic dysfunction or to patients with current or previous episodes of heart failure.^[10]^ Furthermore, AAD is mainly metabolized through liver and kidney and may long-term use might cause drug intolerance for the elderly patients who have liver or renal insufficiency.^[6]^

Traditional Chinese medicine is widely accepted in relieving cardiac arrhythmias symptoms, due to its efficacy. According to previous clinical and experimental investigations, natural drug treatment can greatly reduce the occurrence of cardiac arrhythmias.^[5]^ Shen-Song-Yang-Xin capsule (SSYXC), as a kind of Chinese patent medicine, is now widely used in the treatment of CHD in China. SSYXC was mainly made from red radix salviae miltiorrhiza, red peony root, radix ophiopogonis,ginseng, mfructus schisandrae chinensis, rhizoma coptidis, and glycyrrhizae. A lot of pharmacological studies have shown that SSYXC can effectively reduce oxygen consumption, free radical damage of myocardial cells, improve myocardial ischemia and hypoxia, stimulate blood circulation, increase of coronary blood flow, and improve autonomic nerve function, so as to relieve the symptoms of CHD combined with arrhythmia.^[26]^ In recent years, a number of studies have shown that SSYXC capsules combined with conventional treatment could contribute to coronary artery blood flow increase, platelet aggregation and inhibit fibrinolytic system inhibition, anti-thrombosis, and reduction of occurrence of cardiac arrhythmia.^[23,27]^

However, there is no relevant meta-analysis aiming for the elderly patients and isolated single study has some limitations, such as low quality, small sample size, methodological defect. So it is difficult to evaluate the effective and safety of SSYXC in the treatment of cardiac arrhythmia in elderly patients with CHD. Therefore, this article intends to systematically evaluate the efficacy and safety of SSYXC in treatment of cardiac arrhythmia in elderly patients with CHD based on relevant random control trials published in recent years.

## 2 Methods

### 2.1 Protocol registration

The protocol of this systematic review has been registered in the International Prospective Register of Systematic Reviews (PROS-PERO) registration number CRD42018112570 on October 29, 2018. Systematic review would be conducted referring to this protocol. Any amendment of the protocol will be tracked in PROSPERO if necessary. This protocol is reported in line with both the Preferred Reporting Items for Systematic Review and Meta-Analysis Protocols (PRISMA-P) 2015 statement^[14]^ and the Cochrane Handbook for Systematic Reviews of Interventions.^[11]^

### 2.2 Selection criteria

#### 2.2.1 Types of studies

Randomized controlled trails (RCTs) evaluating the efficacy and safety of SSYXC for treating arrhythmia in senile patient (≥60 years old) with CHD will be included.

#### 2.2.2 Types of participants

CHD patient diagnosed with arrhythmia over age of 60 years and older will be included. The types of arrhythmia include sinus tachycardia or bradycardia, atrial or ventricular premature complex, supraventricular or ventricular tachycardia, atrial flutter or fibrillation, and ventricular fluter or fibrillation. There will be no restriction to sex, age, disease duration, or ethnicity of the participants.

#### 2.2.3 Types of interventions

Patients in control group must have been treated with conventional treatment according to modern guidelines for treating arrhythmia. Patients in experimental group must have been treated with SSYXC together with conventional treatment received by the control group.

#### 2.2.4 Outcomes

##### 2.2.4.1 Primary outcomes

Primary outcomes will include effective rates of treatment and improvements of electrocardiogram or 24 hours dynamic electrocardiogram result.

##### 2.2.4.2 Secondary outcomes

Secondary outcomes will include improvement of relevant serological indexes, heart function classification and adverse events.

### 2.3 Search strategy

Relevant RCTs will be identified and retrieved from databases from their inception to October 2018 including 4 Chinese database (China National Knowledge Infrastructure (CNKI), Chinese VIP Information, Wanfang Database, Chinese Biomedical Database (CBM)), 5 English database (PubMed, Medline, Embase, Cochrane Library, and Web of Science). There will be no restriction on publication type, region, or language for retrieval strategies. The detailed search strategies in PubMed is provided in Table 1.

**Table 1:**
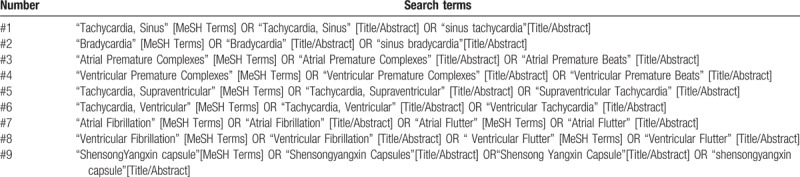
Search strategy for PubMed.

#### 2.3.1 Retrieving other resources

Relevant gray literature will be identified and retrieved by checking the reference lists of the literature retrieved from database. Besides, relevant medical journals will be checked to identify literature not included in electronic databases.

### 2.4 Data collection and analysis

#### 2.4.1 Study selection

**Figure 1:**
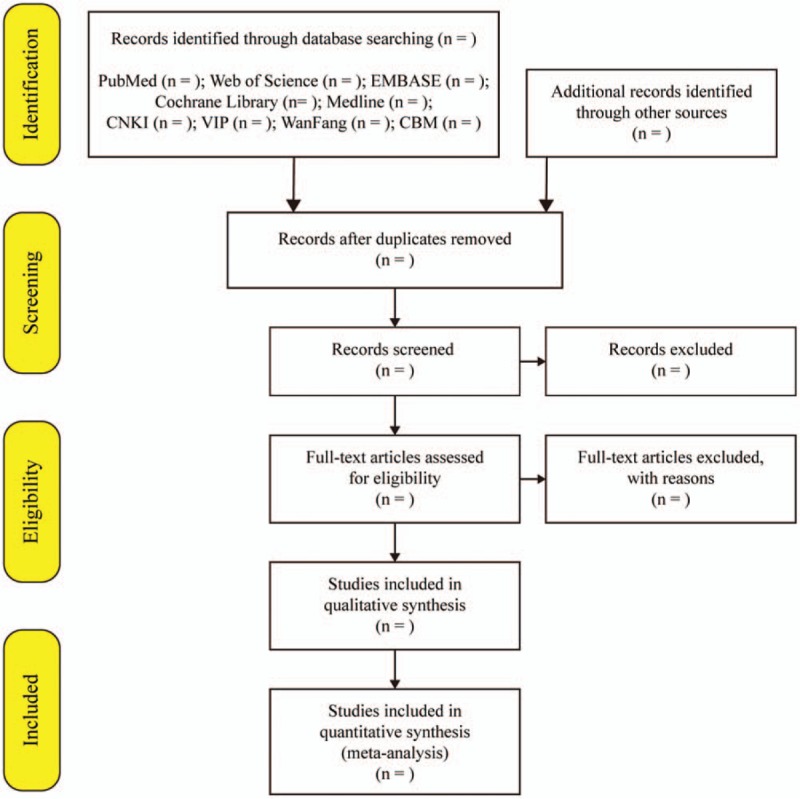
Flowchart of the study selection procedure.

Two independent investigators will conducted the literature search referring to the above search strategies. After removing duplicates, all retrieved literature will evaluated by reading the titles and abstracts and then evaluated their full text to confirmed the eligible studies. Discussion will be conducted if there is any disagreement on study selection. The literature selection process will be reported in accordance with the PRISMA guidelines^[15]^ (Fig. 1).

#### 2.4.2 Data extraction

Data will be extracted and filled in a standardized data collection form by 2 independent investigators. The extracted items will include the first author's name; year of publication; country; sample size; effective rates of treatment group and control group; measures of the intervention; treatment outcomes; and adverse events. Any discrepancies of data extraction will be resolved by discussing with other researchers. If there is any data are insufficient or ambiguous, we will contact the corresponding authors of the included studies through e-mail to obtain additional information

#### 2.4.3 Risk of bias assessment

The methodological quality of the included studies will be assessed by using the Cochrane Collaboration's risk of bias tool.^[12]^ Then, the studies included will be categorized into 1 of 3 groups: “low risk,” “unclear,” or “high risk” after assessing their methodological design such as random sequence generation, allocation concealment, blinding of participants and personnel, blinding of outcome assessments, incomplete outcome data, and selective reporting, etc.^[9]^

Grading of Recommendations Assessment, Development, and Evaluation (GRADE) approach will be employed to evaluate the quality of evidence for each study included.^[17]^ The results of the quality of evidence will be recorded in EXCEL2016 table and shared among researchers via online program GRADEpro (https://gradepro.org/)

Discrepancies will be resolved through discussion among researchers if necessary.

### 2.5 Data synthesis and analysis

The data synthesis and analysis will be performed using Stata 12.0 software and will be shared among researchers in Dropbox (Dropbox, Inc) folders. Details of included studies including participants, interventions, and results will be conducted descriptive analysis respectively. In addition, data of studies using the same types of intervention, comparison, and outcome measure will be conducted a quantitative synthesis.

Data of continuous outcomes will be pooled as the mean difference or standardized mean difference with 95% confidence intervals (CIs) whereas as a risk ratio with 95% CIs for data of dichotomous outcomes.

Heterogeneity of effect measures for studies included will be assessed using both the chi-squared test and the *I*^2^ statistic. *I*^2^ values ≥50% indicated substantial heterogeneity and *I*^2^ values≥75%

When the heterogeneity of studies is significant (*I*^2^ > 75%), a random-effects model will be employed otherwise a fixed-effects model will be employed (*I*^2^ < 75%).

In case of the number of studies included is small or variance estimates of studies have poor accuracy a fixed-effects model will be employed too.

### 2.6 Subgroup analysis

If heterogeneity is evaluated as significant (*I*^2^ > 75%) and the necessary data are available, we will conduct a subgroup analysis to account for the heterogeneity. Subgroup analysis will be conducted providing the heterogeneity of studies is significant (*I*^2^ > 75%). A subgroup analysis will be conducted according to the following criteria: age stratification of patients; the dosage of SSYXC; different type of arrhythmia.

### 2.7 Sensitivity analysis

Sensitivity analyses will be preformed to detect the robustness of our meta-analysis result. Effects of studies with high risks of bias, or missing data, or outliers will be investigated again carefully

### 2.8 Assessment of reporting bias

Funnel plot will be used to assess reporting biases of the studies include. If the funnel plot is asymmetry, it indicated that we will attempt to explain possible reasons. There will be no reporting bias if the funnel plot is symmetry. By contrast, there will be reporting bias if the funnel plot is asymmetry.

### 2.9 Ethics and dissemination

All data included in this study are derived from the published literature and do not include patient personal data, so no ethical approval is required. The final meta-analysis results will be published in a peer-reviewed journal.

## 3 Discussion

CHD is one of the most common cardiovascular diseases worldwide, with the increasing risk of morbidity and mortality.^[18,19]^ Cardiac arrhythmia is also prevalent in China. Study suggests that aging is related to changes in human's cardiac conduction system. In recent years, researches have shown the number of patients with CHD with cardiac arrhythmias is increasing, especially in the elderly patient.^[3,21,22]^ It is undeniable that Western medicine has occupied a crucial position in therapy of cardiovascular disease. However, kinds of Western medicine reportedly have side effects, including potential proarrhythmic effects.^[4,25]^ For elderly patients, plenty of adverse reactions can lead to unsatisfactory prognosis due to their poor physical fitness. To inhibit condition aggravation, researchers are seeking a better therapeutic strategy. A wide application of Chinese patent medicine facilitates the use of SSYXC in treatment of cardiovascular disease. Recent studies have shown that SSYXC has a prominent clinical effect on CHD in elderly patients with cardiac arrhythmia.^[13,24,28]^ Thus, in this article, we will describe the protocol of a proposed study based on the referred Reporting Items for Systematic Reviews and Meta-Analyses guidelines in order to further evaluate the efficacy and safety of SSYXC on CHD in elderly patients with cardiac arrhythmia.

To our knowledge, no systematic review or meta-analysis has been performed to determine the efficacy of SSYXC Capsule on CHD in elderly patients with cardiac arrhythmia. Due to the million effected around the world, this meta-analysis is urgently needed. Our work will be helpful to clinicians in optimizing therapeutic strategies for elderly patients with CHD and cardiac arrhythmia. SSYXC capsule will probably be able to reduce the burden of elderly patients with cardiovascular diseases worldwide. Finally, our work will provide a broader scope of evidence-based research.

## Author contributions

Yinzhi Song conceived the study idea. Zhicong Zeng were responsible for the design of this systematic review. ZhenJie Zhuang contributed to the data analysis plan. YinXian He drafted the manuscript and ZhaoJun Yang edited. All authors provided feedback and approved the final manuscript.

**Conceptualization:** Zhicong Zeng, Yinzhi Song.

**Data curation:** Zhicong Zeng.

**Formal analysis:** YinXian He.

**Methodology:** YinXian He.

**Software:** YinXian He.

**Writing – original draft:** ZhenJie Zhuang.

**Writing – review & editing:** ZhaoJun Yang.

Yinzhi Song orcid: 0000-0001-9220-3785.
